# In vivo and post-mortem measurements of radio frequency induced heating during MRI of pigs implanted with vascular stents

**DOI:** 10.1186/1532-429X-18-S1-P53

**Published:** 2016-01-27

**Authors:** David C Gross, Orlando P Simonetti

**Affiliations:** 1 Biomedical Engineering, The Ohio State University, Columbus, OH USA; 2 Dorothy M. Davis Heart & Lung Research Institute, The Ohio State University, Columbus, OH USA; 3 Radiology, The Ohio State University, Columbus, OH USA; 4 Internal Medicine, Division of Cardiovascular Medicine, The Ohio State University, Columbus, OH USA

## Background

Radio Frequency (RF) induced heating associated with MRI is an increasing safety concern as more patients are implanted with cardiovascular devices. A standard test method (ASTM F2182) is used across the device industry to measure RF heating, but this method does not account for cooling mechanisms such as blood flow and perfusion. We hypothesize that in vivo conditions will significantly lessen the RF induced temperature rise near a medical device. To test our hypothesis we performed in vivo MRI experiments on pigs implanted with vascular stents and temperature probes.

## Methods

Non-survival surgery was performed on 7 anesthetized pigs according to an approved IACUC protocol. Fiber optic probes measured the temperature rise near the distal end of a vascular stent (80 mm long, 7 mm diameter) deployed in the right carotid artery. RF heating experiments were performed using a GRE sequence on a Siemens Tim Trio 3T MRI (TR/TE = 34.7/2 ms, TA = 10 min) with a console SAR of approximately 5.5 W/kg (achieved by overriding the system SAR monitor). The pigs were positioned supine head first and off-center to the left to position the vascular stent where the electric field is known to be high. First, measurements were made with blood flow and perfusion maintained. Then, a balloon was inflated past the distal end of the stent to halt blood flow. Finally, the pig was sacrificed and the RF heating experiment was repeated post-mortem. Additionally, one control pig was implanted with temperature probes in the carotid artery without a stent, and in vivo (with flow) and post-mortem experiments were performed. Multiple measurements were made under each condition in each pig.

## Results

**Figure 1:**
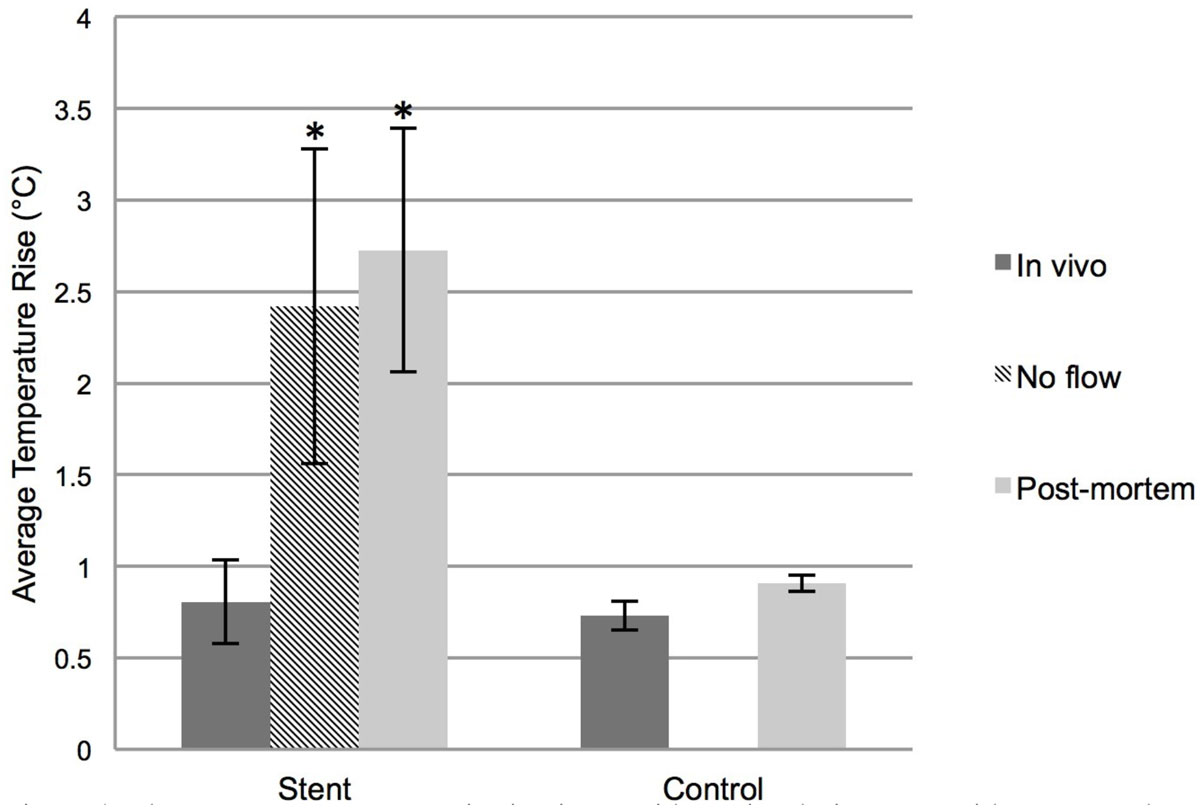
**The average temperature rise in pig carotid arteries during MRI with a stent and without a stent (control) with error bars denoting the standard deviation**. The * denotes a statistically significant difference compared to the control; p < 0.05, using the Steel method for nonparametric comparison with in vivo control. No other significant differences were detected.

Figure 1 shows an average temperature rise of 0.8°C near the distal end of the stent during RF heating in vivo (with blood flow and perfusion, n = 8), 2.4°C in vivo without flow (perfusion without blood flow n = 14) and 2.7°C post-mortem (n = 14). The control experiments in the animal without a stent showed an average temperature rise of 0.7°C in vivo (with blood flow and perfusion, n = 3) and 0.9°C post-mortem (n = 3) in the right carotid artery. The average temperature rise of the stent in the no flow and post-mortem cases were significantly higher (p < 0.05) than the average temperature rise in the in vivo control. The average temperature rise of the stent in vivo (with flow and perfusion) was not significantly different than the in vivo control without the stent.

## Conclusions

Blood flow has a significant cooling effect that reduces the overall temperature rise of a vascular stent during MR-powered RF heating. These results indicate that there are parameters outside the scope of the standard test method that need to be considered when evaluating RF heating. This will lead to more accurate determination of MR device safety, with the goal of ensuring patients with "MR Unsafe" devices are precluded from MRI scans, and those with "MR Conditional" devices have access to MRI scans that may have otherwise been inappropriately withheld.

